# Classic and Atypical Presentations of *Nocardia* Keratitis: A Two‐Case Report Highlighting Diagnostic Challenges

**DOI:** 10.1155/crop/1502013

**Published:** 2026-10-21

**Authors:** Mehrnaz Atighehchian, Zohreh Abedinifar, Alireza Latifi, Roya Jafari, Maryam Kasiri, Ahmad Saberghadim

**Affiliations:** ^1^ Farabi Eye Hospital Tehran University of Medical Sciences Tehran Iran tums.ac.ir; ^2^ Department of Ophthalmology TeMS.C. Islamic Azad University Tehran Iran tiau.ac.ir; ^3^ Microbiology Unit Department of Pathology Tehran University of Medical Sciences Farabi Eye Hospital Tehran Iran tums.ac.ir; ^4^ Department of Medical Parasitology and Mycology School of Public Health Tehran University of Medical Sciences Tehran Iran tums.ac.ir

**Keywords:** case report, in vivo confocal microscopy, infectious keratitis, modified acid-fast staining, *Nocardia* keratitis

## Abstract

*Nocardia* keratitis (NK) is an uncommon infectious keratitis that may present with characteristic or atypical corneal morphology. We report two patients with contrasting clinical appearances and diagnostic courses, highlighting the differing contribution of in vivo confocal microscopy (IVCM) and microbiological testing. A 19‐year‐old male farmer presented after suspected dust exposure with multiple superficial pinhead infiltrates arranged in a wreath‐like pattern. Gram staining showed Gram‐positive branching bacilli, and high‐quality IVCM demonstrated fine hyperreflective branching structures at the ulcer margin. A 37‐year‐old male welder presented after ocular trauma with a sharply demarcated paracentral infiltrate. Initial Gram staining was negative, and an IVCM examination performed at a different site was technically limited and non‐diagnostic; however, culture on chocolate agar grew *Nocardia* species after 3 days, and modified acid‐fast staining confirmed partially acid‐fast branching filaments. Both patients were diagnosed with NK and improved with amikacin‐based topical therapy and sulfacetamide. These cases show that NK may have classic or atypical morphology and that a technically limited or inconclusive IVCM examination does not exclude the diagnosis. Corneal scraping, appropriate staining, and culture remain essential when NK is clinically possible.

## 1. Introduction

*Nocardia* keratitis (NK) is an uncommon infectious keratitis that may mimic fungal or other bacterial ulcers [1, 2]. *Nocardia* species are aerobic, Gram‐positive, weakly acid‐fast branching filamentous bacteria widely found in soil and organic matter [2, 3]. Trauma with dust, soil, or plant material is the leading risk factor, although keratorefractive surgery, corticosteroid use, and contact lens wear have also been described [3–6]. The classic wreath‐like infiltrate may be absent, making diagnosis difficult. The educational value of this report lies in juxtaposing a classic wreath‐like presentation with an atypical sharply demarcated infiltrate in which IVCM was non‐diagnostic. We report these two cases to emphasize the complementary roles and limitations of clinical examination, microbiological testing, and IVCM.

## 2. Case Presentation

### 2.1. Case 1

A 19‐year‐old male farmer presented with 10 days of blurred vision, pain, redness, and photophobia in the left eye after suspected dust exposure. He had no history of contact lens use or prior topical ophthalmic medication. Presenting visual acuity was 0.1 in decimal notation. Slit‐lamp examination showed mild conjunctival hyperemia and multiple superficial patchy pinhead stromal infiltrates measuring approximately 4 × 5 mm, arranged in a peripheral wreath‐like pattern (Figure 1A). The anterior chamber and posterior segment were unremarkable. Corneal scrapings were obtained, and Gram staining demonstrated Gram‐positive branching bacilli (Figure 1B). IVCM was performed at Farabi Eye Hospital using a Heidelberg Retina Tomograph with Rostock Cornea Module (HRT‐RCM; Heidelberg Engineering GmbH, Heidelberg, Germany). The examination showed fine hyperreflective filamentous branching structures, most evident at the active ulcer margins (Figure 2). NK was diagnosed based on the characteristic clinical pattern, smear findings, and supportive IVCM appearance.

**Figure 1:**
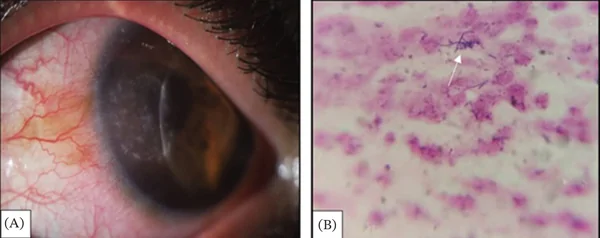
Case 1. (A) Slit‐lamp photograph showing multiple superficial pinhead infiltrates in a wreath‐like pattern. (B) Gram stain showing Gram‐positive branching bacilli.

**Figure 2:**
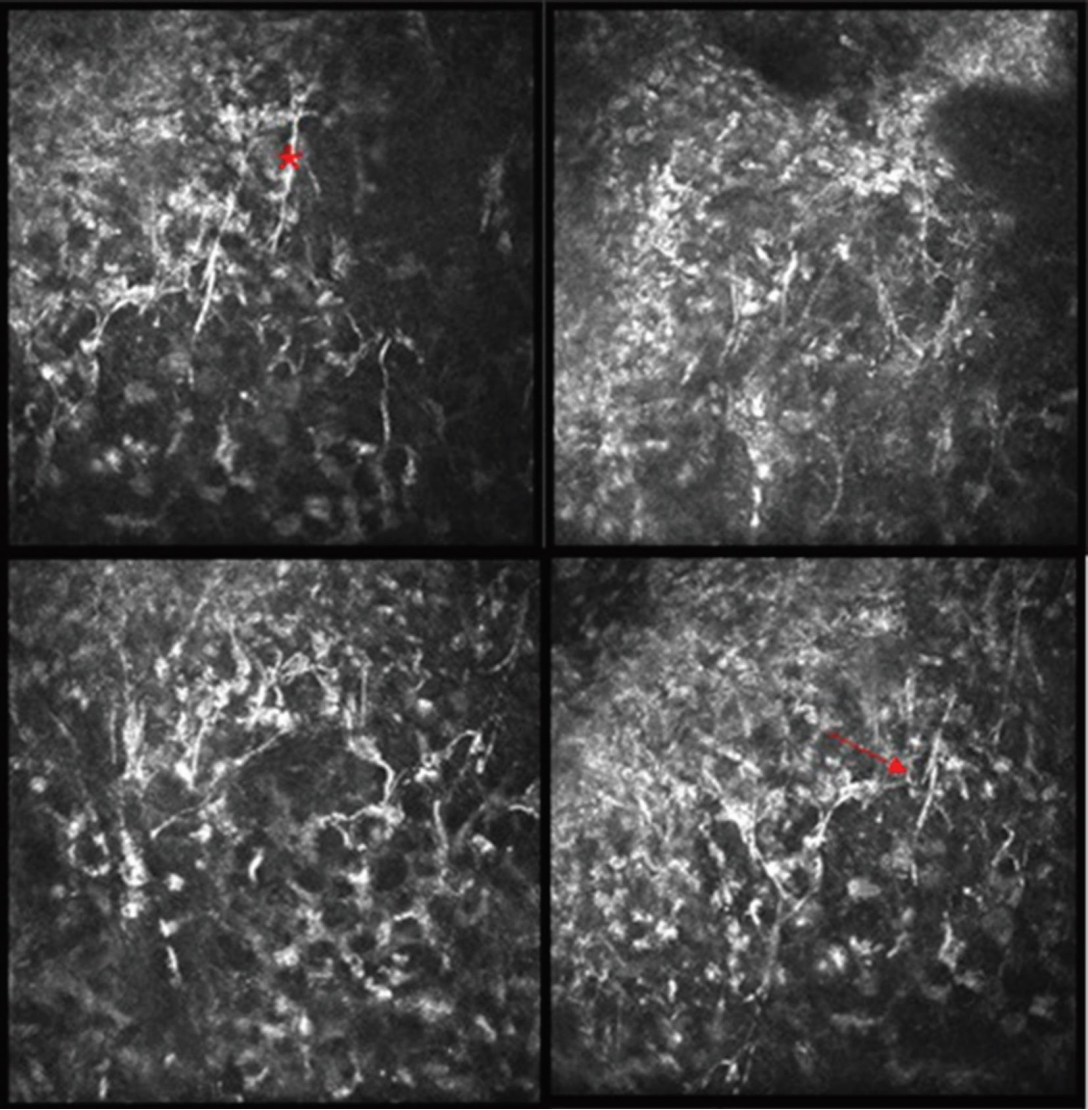
Case 1. In vivo confocal microscopy showing fine hyperreflective filamentous branching structures at the active margin of the corneal ulcer.

Treatment was initiated with topical sulfacetamide 20% every 6 h, fortified topical amikacin hourly, and oral trimethoprim‐sulfamethoxazole 400/80 mg every 12 h. The infiltrates improved without complications during 3 weeks of follow‐up, and best‐corrected visual acuity improved to 1.0 in decimal notation at 2 months.

### 2.2. Case 2

A 37‐year‐old male welder developed pain, redness, and blurred vision in the right eye 8 days after ocular trauma. He had undergone refractive surgery 4 years earlier. Presenting visual acuity was 0.4 in decimal notation. Slit‐lamp examination revealed a 2 × 2 mm superficial gray‐white paracentral corneal infiltrate with sharply demarcated borders and localized edema (Figure 3). Several small whitish foci were visible within the lesion; however, because the available slit‐lamp photograph was suboptimally focused, it was not possible to determine reliably whether these represented the characteristic pinhead infiltrates seen in Case 1. Corneal scraping was performed, but the initial Gram stain was negative. IVCM was performed at the Central Eye Bank of Iran using a different confocal system as part of routine clinical care. The archived report available for this retrospective case did not document the device manufacturer, model, or country of origin, and these details could not be verified retrospectively. The report described hyperreflective stromal haze with inflammatory‐cell infiltration, while the available images were blurred and overexposed, limiting visualization of normal corneal structures and reliable assessment for *Nocardia*‐specific filamentous features (Figure 4). The IVCM examination was therefore considered technically limited and non‐diagnostic rather than negative. *Nocardia* was not initially suspected, and empirical hourly fortified amikacin and cefazolin were started.

**Figure 3:**
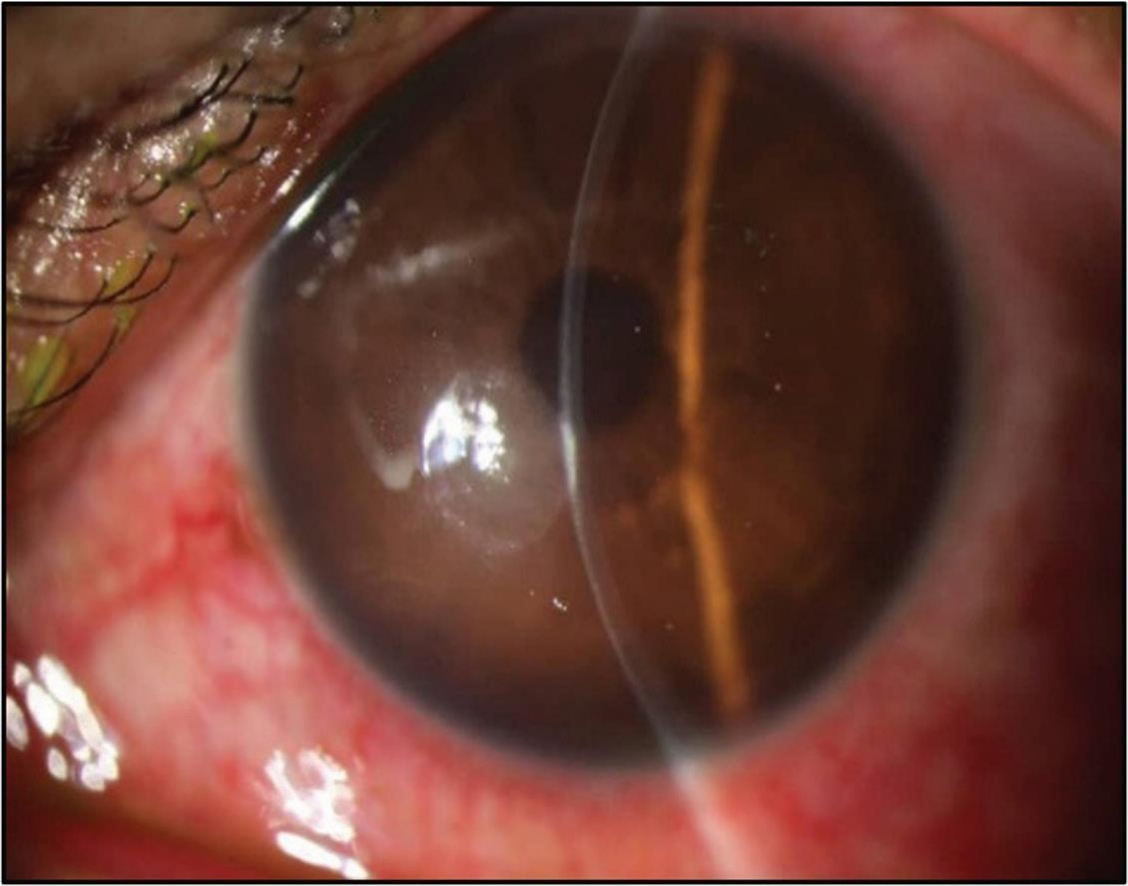
Case 2. Slit‐lamp photograph showing a sharply demarcated superficial gray‐white paracentral infiltrate with localized corneal edema. Several small whitish foci are visible within the lesion, but the image quality precludes confident determination of whether they represent the characteristic pinhead infiltrates seen in Case 1.

**Figure 4:**
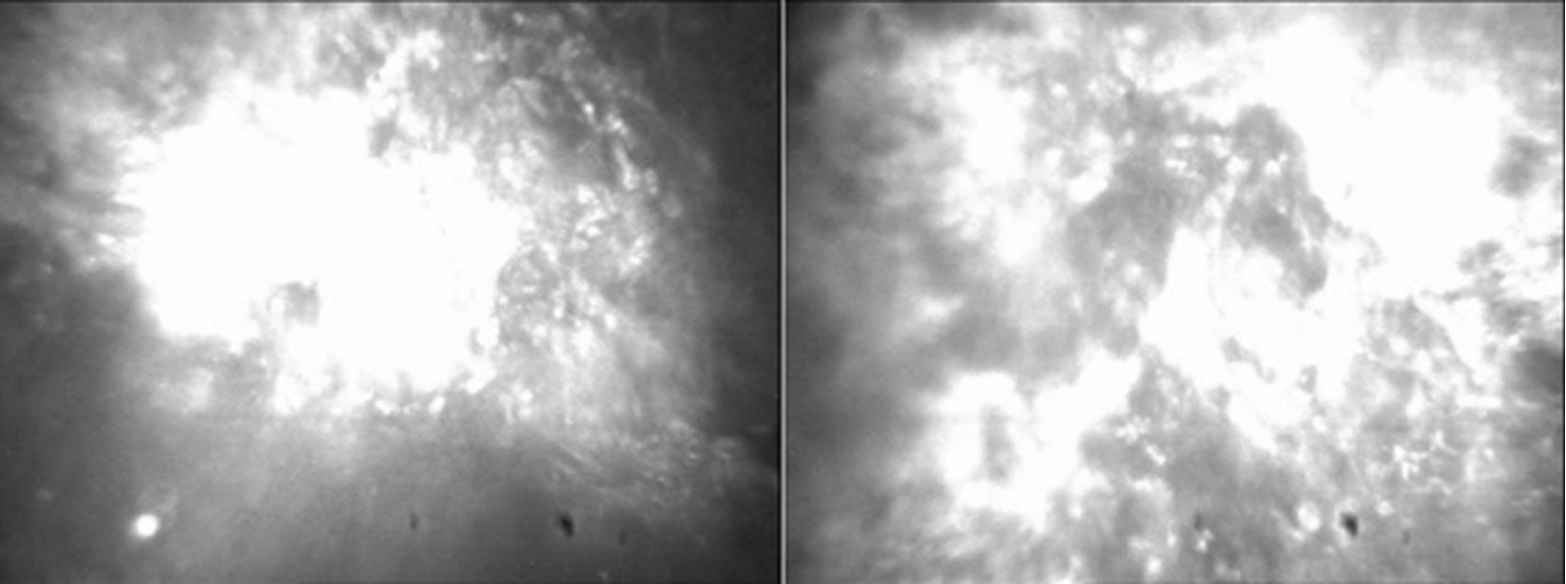
Case 2. In vivo confocal microscopy. The available image is blurred and overexposed, with limited visualization of normal corneal structures and no reliably identifiable *Nocardia*‐specific filamentous structures; the examination was therefore considered technically limited and non‐diagnostic.

After 3 days, dry colonies of *Nocardia* species grew on chocolate agar (Figure 5A). Repeat corneal sampling with modified acid‐fast staining confirmed partially acid‐fast branching filaments (Figure 5B). Treatment was adjusted to topical amikacin and sulfacetamide 20%. After 14 days, the corneal infection improved clinically, and final best‐corrected visual acuity was 0.7 in decimal notation at 4 months. Written informed consent was obtained from both patients for publication of their case details and clinical images.

**Figure 5:**
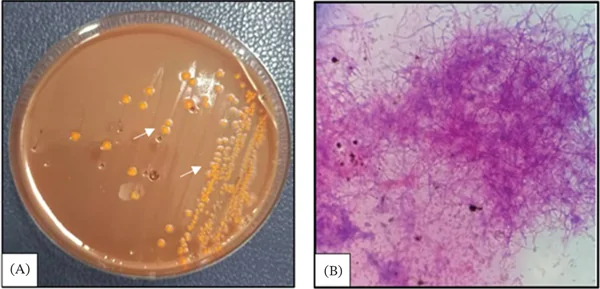
Case 2. (A) Dry colonies of *Nocardia* species on chocolate agar. (B) Modified acid‐fast stain showing partially acid‐fast branching filaments.

### 2.3. Timeline

Case 1: approximately Day −10, ocular symptoms began after suspected dust exposure; Day 0, examination showed a peripheral wreath‐like infiltrate, Gram stain revealed branching Gram‐positive bacilli, and high‐quality IVCM showed branching hyperreflective structures; treatment was initiated on presentation; by 3 weeks, the infiltrates had improved without complications; at 2 months, best‐corrected visual acuity was 1.0 in decimal notation. Case 2: Day −8, ocular trauma occurred followed by pain, redness, and blurred vision; Day 0, a sharply demarcated paracentral infiltrate was identified, Gram stain was negative, and IVCM performed at the Central Eye Bank of Iran was technically limited and non‐diagnostic, prompting empirical fortified amikacin and cefazolin; Day 3, culture grew *Nocardia* species and modified acid‐fast staining confirmed the diagnosis, after which therapy was adjusted to topical amikacin and sulfacetamide 20%; by Day 14, the infection had improved clinically; at 4 months, best‐corrected visual acuity was 0.7 in decimal notation.

## 3. Discussion

These cases illustrate that NK may appear with classic or atypical corneal morphology. The first patient showed the characteristic wreath‐like arrangement of chalky pinhead infiltrates, a pattern considered highly suggestive of NK [3, 7]. The second patient had a small, sharply bordered paracentral infiltrate. Several small whitish foci were visible in the available photograph, but its suboptimal focus precluded confident classification of these foci as the classic pinhead lesions observed in Case 1. The initial Gram stain was negative, and the technically limited IVCM examination was non‐diagnostic. This combination delayed clinical suspicion until culture growth and modified acid‐fast staining established the diagnosis.

Because *Nocardia* can resemble fungal keratitis or other bacterial ulcers, slit‐lamp morphology alone may be insufficient [1, 3]. Gram staining may reveal branching bacilli, and modified acid‐fast staining can support early recognition, but culture remains important and may require 48–72 h [3, 8]. IVCM may help by showing short, thin, branching hyperreflective filamentous structures near the active edge of the infiltrate [9]. In the present report, however, the two IVCM examinations were performed at different sites using different systems and were not acquired under a standardized research protocol. Case 2 images were blurred and overexposed, with poor visualization of regular corneal structures; this technical limitation could reduce diagnostic yield and likely contributed to the non‐diagnostic examination. A technically suboptimal IVCM study should therefore not be interpreted as evidence against NK. When clinical suspicion persists, repeat imaging with optimized focus and exposure, adequate corneal contact, acquisition of multiple high‐quality frames, and targeted sampling of the active infiltrate margin may improve interpretability; microbiological staining and culture remain essential for confirmation [9].

Johansson et al. described a similarly challenging case of NK in which repeated cultures and direct microscopy were initially negative. Later, HRT3‐RCM IVCM demonstrated branching filamentous structures suggestive of *Nocardia*, and subsequent biopsy and optimized culture sampling established the diagnosis. Their isolate was resistant to amikacin, illustrating an additional therapeutic challenge and the importance of susceptibility testing when the clinical response to first‐line therapy is inadequate [10]. Their experience complements the present cases by emphasizing that no single diagnostic modality is sufficient in every patient and that repeated, multimodal evaluation may be necessary when the clinical course is atypical or discordant with initial testing.

Topical amikacin has traditionally been a key treatment for NK [11]. More recent susceptibility data, including the case reported by Johansson et al., have identified amikacin‐nonsusceptible isolates, supporting susceptibility‐guided therapy when culture and sensitivity results are available [8, 10]. In both present cases, amikacin‐based therapy with sulfacetamide was associated with clinical improvement. NK should therefore remain in the differential diagnosis of traumatic keratitis after environmental exposure, even when the infiltrate is not wreath‐like. Early scraping, appropriate staining, culture, and adjunctive IVCM can reduce diagnostic delay and guide targeted antimicrobial treatment.

A strength of this report is the side‐by‐side comparison of two microbiologically confirmed cases with contrasting corneal morphology and different IVCM findings, illustrating both the value and limitations of a multimodal diagnostic approach. Limitations include the two‐patient design, lack of species‐level identification and antimicrobial susceptibility testing, incomplete documentation of the exact post‐culture dosing schedule in Case 2, and non‐standardized IVCM acquisition at different sites using different confocal systems. In addition, the manufacturer, model, and country of origin of the Case 2 IVCM system could not be verified from the archived report. These limitations restrict generalizability, preclude direct comparison of IVCM performance between the two cases, and prevent conclusions about the diagnostic sensitivity of IVCM or comparative treatment efficacy.

## 4. Conclusion

NK should remain in the differential diagnosis of traumatic or environmentally associated infectious keratitis even when the infiltrate lacks the classic wreath‐like appearance. IVCM can provide useful supportive information but cannot exclude NK when findings are negative or inconclusive. Early corneal scraping, appropriate staining, and culture are essential to establish the diagnosis and guide targeted antimicrobial treatment.

## Author Contributions

Mehrnaz Atighehchian: Concept and design; clinical study; data acquisition; manuscript review.

Zohreh Abedinifar: Microbiological investigation; data acquisition; definition of intellectual content; manuscript review.

Alireza Latifi: Microbiological and mycological interpretation; literature search; definition of intellectual content; manuscript review.

Roya Jafari: Clinical study; data acquisition; manuscript editing; manuscript review.

Maryam Kasiri: Data acquisition; literature search; manuscript review.

Ahmad Saberghadim: Concept and design; definition of intellectual content; manuscript editing; manuscript review; final approval; guarantor.

## Funding

No funding was received for this manuscript.

## Disclosure

All authors reviewed and revised the manuscript and take full responsibility for its content.

## Ethics Statement

Ethical approval was not required for this case report in accordance with institutional policy.

## Consent

Written informed consent was obtained from both patients for publication of the case details and accompanying clinical images.

## Conflicts of Interest

The authors declare no conflicts of interest.

## Data Availability

The data that support the findings of this study are available on request from the corresponding author. The data are not publicly available due to privacy or ethical restrictions.

## References

[1] Behaegel J. , Ni Dhubhghaill S. , and Koppen C. , Diagnostic Challenges in Nocardia Keratitis, Eye Contact Lens. (2018) 44, no. 1, S370–S372, 10.1097/ICL.0000000000000462, 29219900. 29219900

[2] Sridhar M. S. , Gopinathan U. , Garg P. , Sharma S. , and Rao G. N. , Ocular Nocardia Infections With Special Emphasis on the Cornea, Survey Ophthalmology. (2001) 45, no. 5, 361–378, 10.1016/S0039-6257(00)00207-1, 11274691. 11274691

[3] Sridhar M. S. , Sharma S. , Reddy M. K. , Mruthyunjay P. , and Rao G. N. , Clinicomicrobiological Review of Nocardia Keratitis, Cornea. (1998) 17, no. 1, 17–22, 10.1097/00003226-199801000-00003, 9436875. 9436875

[4] Garg P. , Sharma S. , Vemuganti G. K. , and Ramamurthy B. , A Cluster of Nocardia Keratitis After LASIK, Journal of Refractive Surgery. (2007) 23, no. 3, 309–312, 10.3928/1081-597X-20070301-17, 17385300. 17385300

[5] Javadi M. M. , Kanavi M. M. , Zarei M. S. , Mirbabaei M. F. , Jamali M. H. , Shoja M. M. , Mahdavi M. M. , Naghshgar M. N. , Yazdani M. S. , and Faramarzi M. A. , Outbreak of Nocardia Keratitis After Photorefractive Keratectomy: Clinical, Microbiological, Histopathological, and Confocal Scan Study, Journal of Cataract & Refractive Surgery. (2009) 35, no. 2, 393–398, 10.1016/j.jcrs.2008.08.045, 19185261. 19185261

[6] Lalitha P. , Tiwari M. , Prajna N. V. , Gilpin C. , Prakash K. , and Srinivasan M. , Nocardia Keratitis, Cornea. (2007) 26, no. 3, 255–259, 10.1097/ICO.0b013e318033d853. 17413948

[7] Rao S. K. , Madhavan H. N. , Sitalakshmi G. , and Padmanabhan P. , Nocardia Asteroides Keratitis: Report of Seven Patients and Literature Review, Indian Journal of Ophthalmology. (2000) 48, no. 3, 217–221, 11217254. 11217254

[8] Adre E. , Maestre-Mesa J. , Durkee H. , Arboleda A. , Flynn H. W. , Amescua G. , Parel J. M. , and Miller D. , Nocardia Keratitis: Amikacin Nonsusceptibility, Risk Factors, and Treatment Outcomes, Journal of Ophthalmic Inflammation and Infection. (2022) 12, no. 1, 10.1186/s12348-022-00287-1, 35247126. PMC8898206 35247126

[9] Vaddavalli P. K. , Garg P. , Sharma S. , Thomas R. , and Rao G. N. , Confocal Microscopy for Nocardia Keratitis, Ophthalmology. (2006) 113, no. 9, 1645–1650, 10.1016/j.ophtha.2006.03.054, 16828508. 16828508

[10] Johansson B. , Fagerholm P. , Petranyi G. , Claesson Armitage M. , and Lagali N. , Diagnostic and Therapeutic Challenges in a Case of Amikacin-Resistant Nocardia Keratitis, Acta Ophthalmology. (2017) 95, no. 1, 103–105, 10.1111/aos.13182, 27572657. 27572657

[11] Denk P. O. and Thiel H. J. , Amikacin May be Drug of Choice in Nocardia Keratitis, British Journal of Ophthalmology. (1996) 80, no. 10, 928–929, 10.1136/bjo.80.10.928, 8976710. PMC505655 8976710

